# The distribution of heterophilic antigens and their relationship with autoimmune diseases

**DOI:** 10.3389/fimmu.2023.1275658

**Published:** 2023-11-10

**Authors:** Lijun Sun, Nana Wang, Yangmeng Feng, Xueping Huo, Qing Feng, Xiangrong Zhao, Yan Li, Liting Yan, Xin Xie, Jun Hu

**Affiliations:** ^1^Shaanxi Provincial Key Laboratory of Infection and Immune Diseases, Shaanxi Provincial People’s Hospital, Xi’an, Shaanxi, China; ^2^Shaanxi Province Research Center of Cell Immunological Engineering and Technology, Xi’an, Shaanxi, China; ^3^Key Laboratory of Resource Biology and Biotechnology in Western China, Ministry of Education, College of Life Sciences, Northwest University, Xi’an, Shaanxi, China

**Keywords:** heterophilic antigen, heterophilic antibody, autoimmune reaction, carrier effect, microorganisms

## Abstract

**Introduction:**

Microbial infections are associated with the occurrence of autoimmune diseases, but the mechanisms of microbial infection inducing autoimmune diseases are not fully understood. The existence of heterophilic antigens between microorganisms and human tissues may explain part of the pathogenesis of autoimmune diseases. Here, we investigate the distribution of heterophilic antigens and its relationship with autoimmune diseases.

**Methods:**

Monoclonal antibodies against a variety of microorganisms were prepared. The titer, subclass and reactivity of antibodies with microorganisms were identified, and heterophilic antibodies that cross-reacted with human tissues were screened by human tissue microarray. The reactivity of these heterophilic antibodies with different individuals and different species was further examined by immunohistochemistry.

**Results:**

In this study, 21 strains of heterophilic antibodies were screened. The results showed that these heterophilic antibodies were produced due to the existence of heterophilic antigens between microorganism and human body and the distribution of heterophilic antigens had individual, tissue and species differences.

**Conclusion:**

Our study showed that heterophilic antigens exist widely between microorganisms and human body, and the heterophilic antigens carried by microorganisms may break the immune tolerance of the body through carrier effect and initiate immune response, which may be one of the important mechanisms of infection inducing autoimmune diseases.

## 1 Introduction

Autoimmune diseases (AID) are a group of diseases that result from damage to one’s own organs caused by the action of immune cells or immune reaction products on its own antigens (1). The pathogenesis of AID is not fully understood, neither it’s clear how the immune response that attacks itself is initiated. In 1962, Kaplan and Meyeserian found that rabbit serum immunized with Group A streptococcus antigen cross-reacted with human heart tissue, providing the first evidence of the correlation between microbial infection and autoimmune reaction (2). Further studies showed that the cross-reactive antibodies were stimulated by antigens shared by Group A hemolytic streptococcus and human heart tissue (3). Subsequently, more and more microorganisms were found to share antigens with human tissues, and such shared antigens between microorganisms and human tissues were known as heterophilic antigens (4–7).

The presence of heterophilic antigens between microorganisms and human tissues may explain part of the pathogenesis of autoimmune disease, but research on heterophilic antigens is currently scattered. At the same time, heterophilic antigens are always present in the body. But why they do not elicit an autoimmune response. And when that heterophilic antigen is carried by a microorganism, it may stimulate the body to produce the immune responses. How heterophilic antigens break through the immune tolerance in the body has not been elucidated.

In this study, we immunized Balb/c mice with a variety of pathogenic microbial and prepared hundreds of monoclonal antibodies. 21 strains of monoclonal antibodies were found to cross-react with normal human tissues, indicating that heterophilic antigens exist widely between pathogenic microorganisms and human tissues. Further studies have found that the distribution of heterophilic antigens is different in distinct tissues, individuals and species. These findings can significantly improve our understanding, prevention and treatment of autoimmune diseases. We also found that monoclonal antibodies produced by heterophilic antigens carried by pathogenic microorganisms could also react with tissue antigens in mice, suggesting that the carrier effect plays an important role in the mechanism of infection-induced autoimmune diseases.

## 2 Materials and methods

### 2.1 Materials

The H1N1 influenza virus split vaccine was purchased from Hualan Biological Bacterin Co., Ltd. The H5N1 avian influenza virus inactivated vaccine was purchased from Yebio Engineering Co., Ltd. The H7N9 avian influenza virus inactivated vaccine was donated by the Chinese Center for Disease Control and Prevention. Respiratory Syncytial Virus antigen (RSV, EL-07-03), Adenovirus Grade 2 Hexon protein (AD, EL-15-02), Parainfluenza virus Type 2 Grade 2 antigen (PARA, EL-09-02), Chlamydia pneumoniae Cell Lysate (CP, EL-46-02) and EBV Capsid antigen (EL-16-07) were purchased from Microbix, USA. RPMI1640 medium was purchased from Hyclone, fetal bovine serum (FBS) was purchased from Hangzhou Sijiqing, HRP-labeled goat anti-mouse secondary antibody and immunohistochemical staining kit were purchased from Beijing Zhongshan Jinqiao Biotechnology Co., LTD. Fluorescein isothiocyanate (FITC)-conjugated goat anti-mouse IgG and the fluorescent dye DAPI were purchased from Invitrogen (Grand Island, NY).

Two normal human tissue chips, one containing 33 different tissues or organs, the other containing 21 different people of the same tissue or organ were purchased from Shaanxi Chaoying Biotechnology Co., LTD. The normal human tissue chip contains 33 different tissues and organs, including the brain, cerebellum, peripheral nerves, adrenal glands, thyroid glands, spleen, thymus, bone marrow, lymph nodes, tonsils, pancreas, liver, esophagus, stomach, small intestine, colon, lungs, tongue, throat, kidneys, bladder, testicles, prostate, penis, ovaries, fallopian tubes, mammary gland, uterus, cervix, heart, eyes, striated muscles, and skin.

### 2.2 Preparation and characterization of monoclonal antibodies

The monoclonal antibodies were prepared by reference to previously reported methods (8). Briefly, mice were first immunized with Freund complete adjuvant and vaccines or antigens emulsified in a 1:1 ratio. After 21 days, the mice were injected subcutaneously with the same dose of the Freund incomplete adjuvant and antigens to boost immunity. Seven days later, the mice were intraperitoneally injected with the antigens. On the third day before preparation for fusion, mice were injected intraperitoneally with proteins to boost immunity. The spleen cells of immunized mice were fused with myeloma SP2/0 cells at a ratio of 10:1. When the hybridoma cells reached 20-50%, the positive cells were screened by ELISA method. The positive holes were cloned by limited dilution method, and the specific antibody-secreting cell line was obtained after 3 times of cloning, which was cultured and cryopreserved. The graded diluent of monoclonal antibodies were prepared and the titer of monoclonal antibodies were detected by ELISA method. SP2/0 was used as negative control. SBA Clonotyping™ System/HRP kit was used to identify the subclasses of antibodies according to the instructions.

### 2.3 Screening for heterophilic monoclonal antibodies

The microarray containing 33 normal human tissues and immunohistochemical staining were used to detect the binding properties of various antimicrobial monoclonal antibodies to normal human tissues. Briefly, the tissue chips were dewaxed, washed with water, and treated with 3% H_2_O_2_ to block endogenous peroxidase for 30 min at room temperature. Then, pH6.0 sodium citrate buffer was used for microwave repair for 10 min. After 30 min of treatment with normal goat serum, the excess serum was removed and monoclonal antibodies with appropriate dilution were added overnight at 4°C. HRP labeled goat anti-mouse secondary antibody was added for 37°C for 30 min and color development was performed by DAB for 10 min. Finally, it was restained with hematoxylin, dehydrated, transparent, sealed and observed under microscope.

### 2.4 Identification of heterophilic monoclonal antibodies

#### 2.4.1 Immunofluorescence

Influenza virus A/PR8/34 (H1N1) was used to infect MDCK cells. Sp2/0 was used as a negative control. 24h after infection, the cells were fixed with formaldehyde for 20 min. Then goat serum was added and the samples were sealed for 30 min. Diluted monoclonal antibodies H1-13 and H1-84 were added and incubated at 37°C for 2h, FITC-labeled fluorescent secondary antibodies were treated at 37°C for 1h, and DAPI was added to stain for 30 min. Finally, the samples were observed and photographed under the BX41 fluorescence microscope (Olympus Corporation). Images were captured via Image-Pro Plus analysis software 6.0 (Media Cybernetics).

#### 2.4.2 Western blotting

Different viral antigens were subjected to SDS-PAGE electrophoresis. After the electrophoresis, the gels were removed, the protein bands in the gels were transferred to the nitrate cellulose membrane, and then closed with 5% skim milk for 1h. Monoclonal antibodies including H7N9-59, H7N9-73, H7N9-79, H5N1-32, H5N1-63, H5N1-67, EBV-1, EBV-5, EBV-7 and ADV-6 were incubated with corresponding antigens at 4°C overnight, respectively. HRP labeled goat anti-mouse antibody was used as secondary antibody. Chemiluminescence was used to produce color, and finally the images were taken with a gel imaging system.

### 2.5 Reactions of heterophilic antibodies with different pathogenic microorganisms

The reactivity of 21 strains of heterophilic antibodies with different pathogenic microorganisms were evaluated by indirect ELISA. Briefly, the 96−well plate was pre−coated with 100 µl of each antigen (2−5 µg/ml). After washing three times with PBST, the plates were blocked with 200 µl skim milk and incubated for 1 h at 37°C. Subsequently, 100 µl/well supernatant for antibodies was added, including the supernatant of SP2/0 as a negative control, which was incubated for 1 h at 37°C. After washing a further three times, HRP−labeled goat−anti−mouse IgG was added and incubated for 1 h at 37°C. Next, 100 µl TMB−H_2_O_2_ chromogenic solution was added to each well and incubated for 10 min at 37°C in the dark, and terminated with H_2_SO_4_ solution. Finally, the proportion of bound antibodies was measured with an ELISA reader via absorbance at 450 nm. The ratio of each test sample (test sample OD 450 to negative control OD 450) was calculated. Samples with a ratio of ≥2.5 were classified as exhibiting a positive reaction.

### 2.6 Reactions of heterophilic antibodies with different individuals

10 strains of heterophilic antibodies were selected, including monoclonal antibodies H1-84, H5-67, H7N9-58, H7N9-59 reacting with human pancreas, monoclonal antibodies ADV-6, H7N9-77, H7N9-78, H7N9-79 reacting with human stomach, and monoclonal antibodies ADV-6, CP-1 reacting with human kidney. Three kinds of tissue chips containing 21 different human stomach, kidney and pancreas were stained by immunohistochemical method to observe the reaction between heterophilic antibodies and different individuals.

### 2.7 Reactions of heterophilic antibodies with different species

14 strains of antibodies were selected, including monoclonal antibodies ADV-6, CP-1 and H5-32 reacting with human kidney tissue, monoclonal antibodies H7N9- 71, H7N9-73, H7N9-77, H7N9-78, H7N9-79 and H5-32 reacting with human stomach, and monoclonal antibodies H1-17, H1-55, H5-67, H7N9-58, H7N9-59 reacted with human pancreas. Immunohistochemical staining of the above monoclonal antibodies were performed with different animal tissues.

### 2.8 Serum blocking experiment of patients infected with Epstein-Barr virus

#### 2.8.1 Detection of heterophilic antibodies

60 sera from patients with clinical EBV infection were collected and tested for anti-EBV antibody potency by ELISA. 5 sera with antibody potency above 10^-3^ were selected and then immunohistochemical analysis was performed on these 5 sera using human normal tissue microarrays to detect whether these sera produced heterophilic antibodies that could bind to normal human skin, testis or esophageal tissues.

#### 2.8.2 Blocking experiment

The tissue chips were dewaxed, washed with water, and treated with 3% H_2_O_2_ to block endogenous peroxidase for 30 min at room temperature. Then, pH6.0 sodium citrate buffer was used for microwave repair for 10 min. After 30 min of treatment with normal goat serum, the excess serum was removed and positive patient serum (10%) or negative patient serum (10%) were added separately for 1 h at 37°C. EBV-5 monoclonal antibody (1:2000) was added overnight at 4°C. HRP labeled goat anti-mouse secondary antibody was added for 37°C for 30 min and color development was performed by DAB for 10 min. Finally, it was restained with hematoxylin, dehydrated, transparent, sealed and observed under microscope.

## 3 Result

### 3.1 Screening for heterophilic monoclonal antibodies

**Figure 1:**
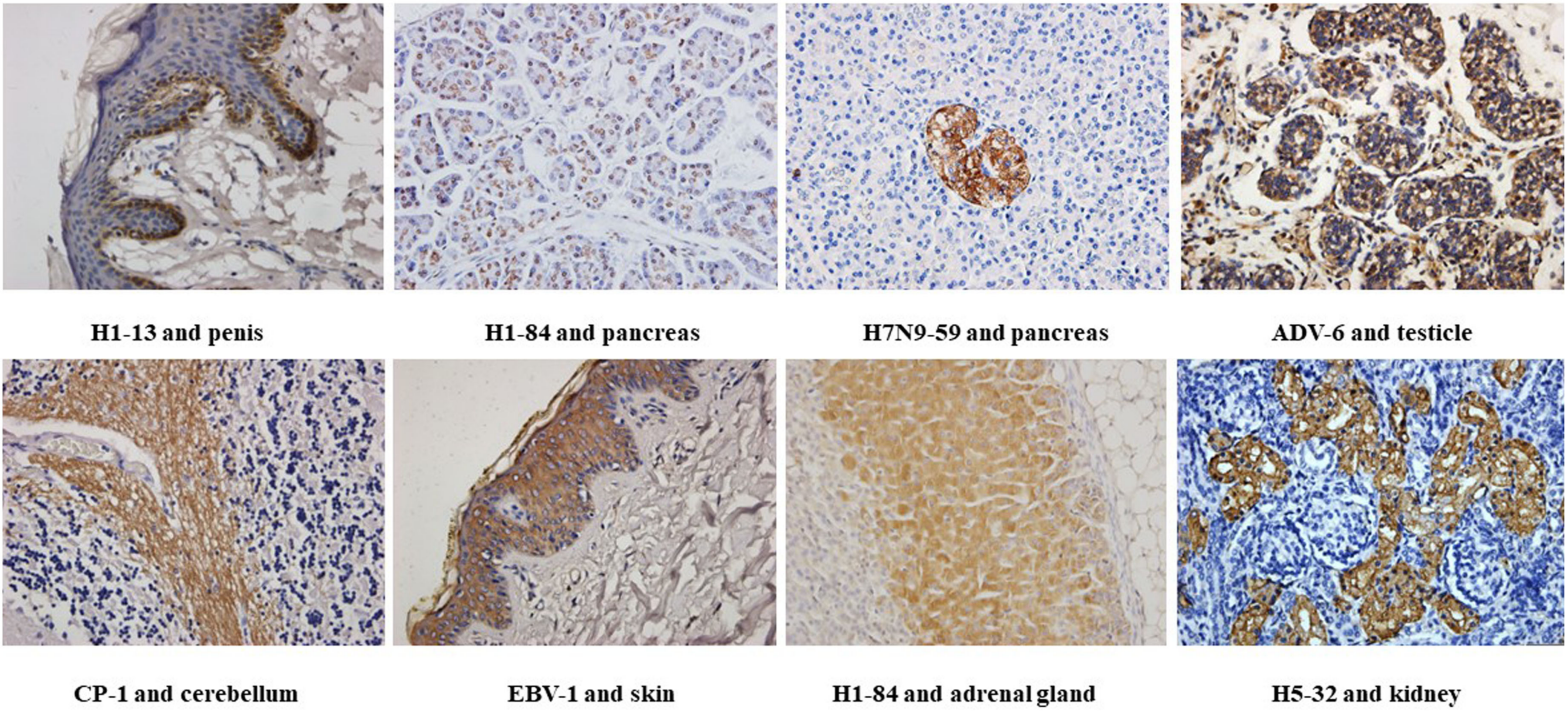
Immunohistochemical detection of the binding of different heterophilic antibodies to different tissues of the human body: magnification of the image is 400x.

We used tissue chips containing 33 different human organs (each organ containing two tissues, 33 organs with a total of 66 tissue microarrays) for immunohistochemical staining. 268 strains of monoclonal antibodies against various pathogenic microorganisms such as H1N1, H5N1 and H7N9 were tested with tissue chips respectively, and 21 strains of antibodies were screened for cross-reaction with human tissues (**Table 1**). Further, the cross-reaction between these 21 antibodies and tissues were verified by immunohistochemistry using other human tissue sections. Some of the immunohistochemistry results are shown in **Figure 1**. These results indicated that heterophilic antigens were widely present between various pathogenic microorganisms and human tissues.

**Table 1:** Prepared monoclonal antibodies against various microorganisms and screened heterophilic antibodies.

Microorganisms	mAbs	Heterophilic mAbs
H1N1	99	4
H5N1	69	3
H7N9	59	7
RSV	5	1
AD	10	1
PARA	8	1
EBV	13	3
CP	5	1
Total	268	21

### 3.2 Characterization of heterophilic monoclonal antibodies

#### 3.2.1 General properties of heterophilic monoclonal antibodies and cross-reactivity with other pathogenic microorganisms

The titers, subclasses and reactivity of heterophile monoclonal antibodies were determined by ELISA method. The titers of 21 monoclonal antibodies were all ≥1:1000. The subclasses of antibodies and their reactivity with pathogenic microorganisms were shown in **Table 2**. The results showed that all of the 21 strains of heterophilic monoclonal antibodies reacted with the corresponding pathogens, and at the same time, some of the heterophilic antibodies not only reacted with the corresponding pathogens, but also cross-reacted with other pathogens. These results suggested that heterophilic antigens were cross-distributed among different pathogenic microorganisms.

**Table 2:** Characteristics of the cross-reactivity of mAbs.

mAb	Titer	Ig subtype	H1N1	H5N1	H7N9	ADV	RSV	CP	PARA2	EBV
H1N1-13	10^5^	IgG1	+							
H1N1-17	10^4^	IgM	+	+	+	+				
H1N1-55	10^3^	IgG1	+				+			
H1N1-84	10^4^	IgM	+	+						
H5N1-32	10^6^	IgM	+	+	+	+				
H5N1-63	10^6^	IgG1	+	+		+				
H5N1-67	10^4^	IgG1	+	+			+	+		+
H7N9-58	10^5^	IgM	+	+	+					
H7N9-59	10^5^	IgM	+	+	+		+			
H7N9-71	10^4^	IgM	+	+	+		+			
H7N9-73	10^5^	IgM	+	+	+					
H7N9-77	10^3^	IgM	+	+	+					
H7N9-78	10^4^	IgM	+	+	+			+		
H7N9-79	10^5^	IgM	+	+	+					
AD-6	10^5^	IgG1	+	+		+	+	+		
RSV-2	10^3^	IgG1					+			
EBV-1	10^6^	IgG1	+			+	+			+
EBV-5	10^5^	IgG1				+				+
EBV-7	10^6^	IgG1	+			+	+			+
PARA-8	10^6^	IgG1				+	+		+	
CP-1	10^3^	IgG1	+	+			+	+	+	+

+, Positive.

#### 3.2.2 Immunofluorescence and western blotting to identify heterophilic monoclonal antibodies

**Figure 2:**
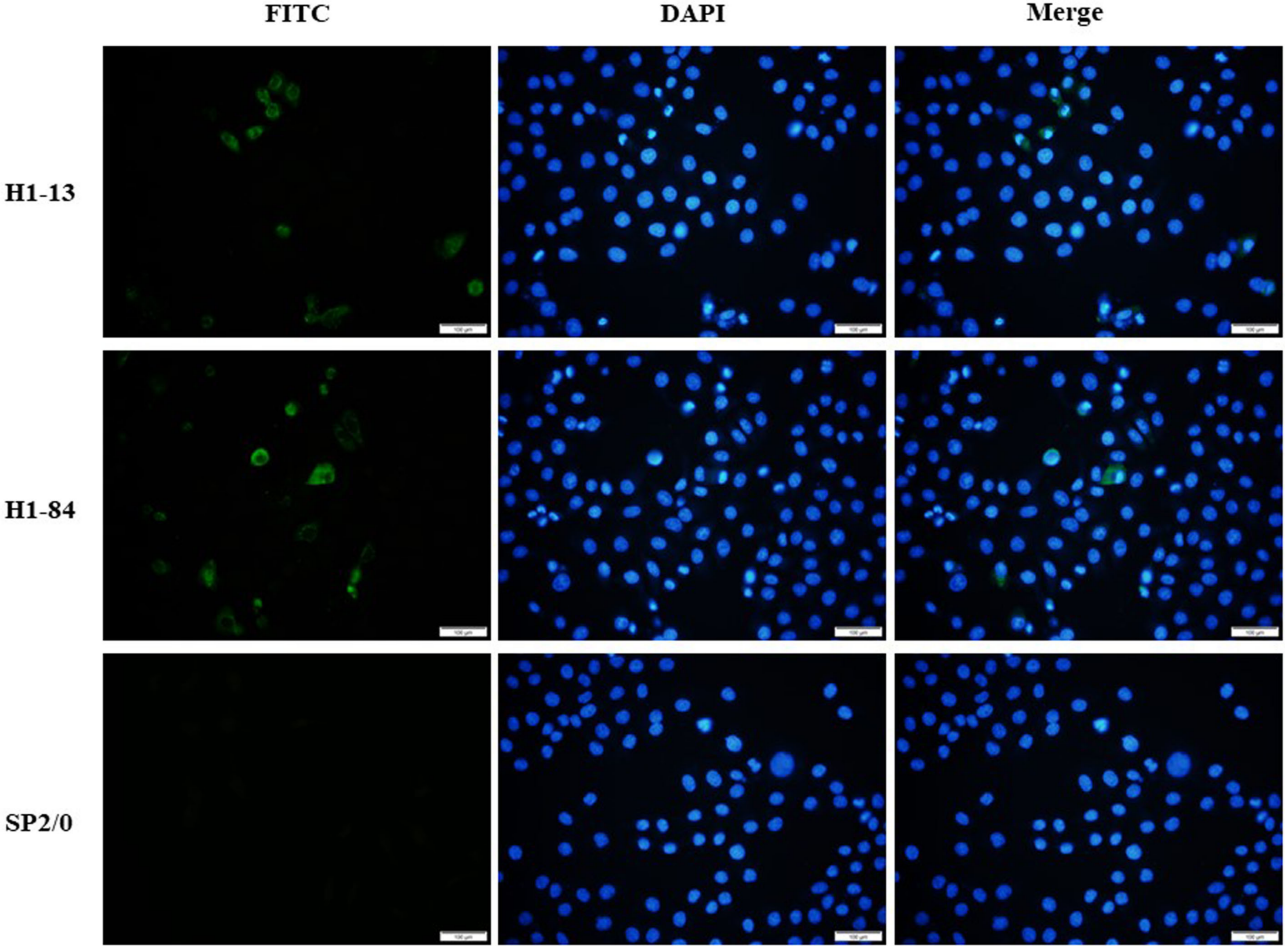
Immunofluorescence detection of heterophilic antibodies binding to virus-infected cells: the first column shows FITC staining of virus-infected cells, the second column shows DAPI staining of nuclei, and the third column shows a combination plot. Sp2/0 was used as a negative control. Magnification of the image is 200x.

Immunofluorescence staining experiments were performed using virus-infected cells with the corresponding antibodies. The results showed that monoclonal antibodies H1-13 and H1-84 reacted specifically with H1N1-infected MDCK cells, while SP2/0 cell culture supernatant did not react with H1N1-infected MDCK cells, indicating that the antibodies H1-13 and H1-84 were derived from the antigen of the influenza virus and recognized the natural proteins of the H1N1 influenza virus (**Figure 2**).

**Figure 3:**
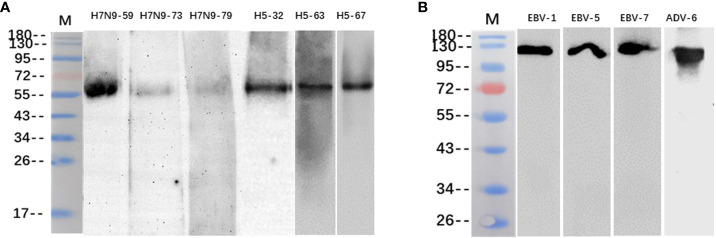
Identification of heterophilic antibodies by Western blotting: **(A)** M, Marker; columns 1-3 show antibodies H7N9-59, H7N9-73 and H7N9-79 reacting with HA antigen of H7N9 influenza virus; column 4-6 shows antibodies H5N1-32, H5N1-63 and H5N1-67 reacting with HA antigen of H5N1 influenza virus. **(B)** M, Marker; columns 1-3 show antibodies EBV-1、EBV-5 and EBV-7 reacting with EBV capsid antigen; column 4 shows antibody ADV-6 reacting with adenovirus hexon antigen.

The binding of some heterophilic monoclonal antibodies to the corresponding target antigens of pathogenic microorganisms was detected by western blotting, and the results showed that the antibodies H7N9-59, H7N9-73 and H7N9-79 against H7N9 influenza virus reacted specifically with the HA protein of H7N9 influenza virus respectively (55KD); the antibodies H5N1-32, H5N1-63 and H5N1-67 against H5N1 influenza virus reacted specifically with the HA protein of H5N1 influenza virus respectively (55KD); the antibodies EBV-1, EBV-5 and EBV-7 reacted with the capsid antigen of EB virus respectively (125KD); the antibody ADV-6 against adenovirus reacted with the hexon antigen of the adenovirus (116KD) (**Figure 3**).

### 3.3 The distribution of heterophilic antigens has tissue differences

The results in **Table 3** show that 21 heterophilic monoclonal antibodies bound to a total of 17 human tissues. Some of these monoclonal antibodies bound to a single human tissue, for example, H1-13 bound to human penile tissue and H7N9-58 bound to human islet tissue, indicating that there were heterophilic antigens between penis tissue and islet tissue and influenza virus, respectively. Some monoclonal antibodies bound to two or more tissues, for example, H5-32 can react with normal human stomach and kidney tissues; H5-63 can react not only with normal human small and large intestine tissues, but also with lung tissues; H1-84 can bind to human cerebellum, adrenal gland, pancreas, testis and other tissues, indicating that the same or similar heterophilic antigens can be distributed on different tissues. These results suggested that the tissue distributions of the heterophilic antigens bound by these heterophilic monoclonal antibodies were variable in the human body.

**Table 3:** Reactivity of the mAbs to normal human tissues.

mAb	Cer	Ag	Tonsil	Pa	Eso	Sto	Si	Colon	lung	Kidney	Bladder	Tes	Penis	Ovary	Mg	Sm	Skin
H1N1-13													+				
H1N1-17				+									+				
H1N1-55				+									+				
H1N1-84	+	+		+								+	+				
H5N1-32						+				+			+				
H5N1-63							+	+	+				+	+	+		
H5N1-67				+									+				
H7N9-58				+													
H7N9-59				+							+						
H7N9-71						+											
H7N9-73						+											
H7N9-77						+											
H7N9-78						+											
H7N9-79						+											
AD-6			+		+	+				+		+				+	
RSV-2				+													
EBV-1					+												+
EBV-5												+					
EBV-7					+												+
PARA-8					+												
CP-1	+									+		+					

Cer(Cerebellum) Ag(Adrenal gland) Pa(Pancreas) Eso(Esophagus) Sto(Stomach) Si(Small intestine) Tes(Testicle) Mg(Mammary gland) Sm(Striated muscle).

+, Positive.

### 3.4 The distribution of heterophilic antigens has individual differences

Immunohistochemical staining was performed on 10 strains of heterophilic antibodies and 21 different human stomach, pancreas and kidney tissue chips, respectively. The results showed that the distribution of binding antigens of each strain of antibodies was different in the population. The proportions of monoclonal antibodies against H5-67, H1-84, H7N9-58 and H7N9-59 were 9.5%, 38.1%, 80.9% and 71.4% with 21 human pancreatic tissue chips, respectively. The proportions of antibodies against ADV-6, H7N9-77, H7N9-78 and H7N9-79 combined with 21 human stomach tissue chips were 47.6%, 47.6%, 66.7% and 61.9%, respectively. The proportions of monoclonal antibodies against ADV-6 and CP-1 binding to 21 human kidney tissue chips were 19.0% and 42.8%, respectively (**Table 4**). These results suggested that the distributions of heterophilic antigens in different individuals were different, which may be the reason why some people get sick and others do not get sick when infected with the same pathogenic microorganism.

**Table 4:** Reactivity of the mAbs to 21 normal human tissues.

	Positive (%)	Negative (%)	Total
Antibodies react with pancreatic tissue			
H5-67	2 (9.5%)	19 (90.5%)	21
H1-84	8 (38.1%)	13 (61.9%)	21
H7N9-58	17 (80.9%)	4 (19.1%)	21
H7N9-59	15 (71.4%)	6 (28.6%)	21
Antibodies react with stomach tissue			
H7N9-77	10 (47.6%)	11 (52.4%)	21
H7N9-78	14 (66.7%)	7 (33.3%)	21
H7N9-79	13 (61.9%)	8 (38.1%)	21
AD-6	10 (47.6%)	11 (52.4%)	21
Antibodies react with kidney tissue			
AD-6	4 (19.0%)	17 (81.0%)	21
CP-1	9 (42.8%)	12 (57.2%)	21

### 3.5 The distribution of heterophilic antigens has species differences

**Figure 4:**
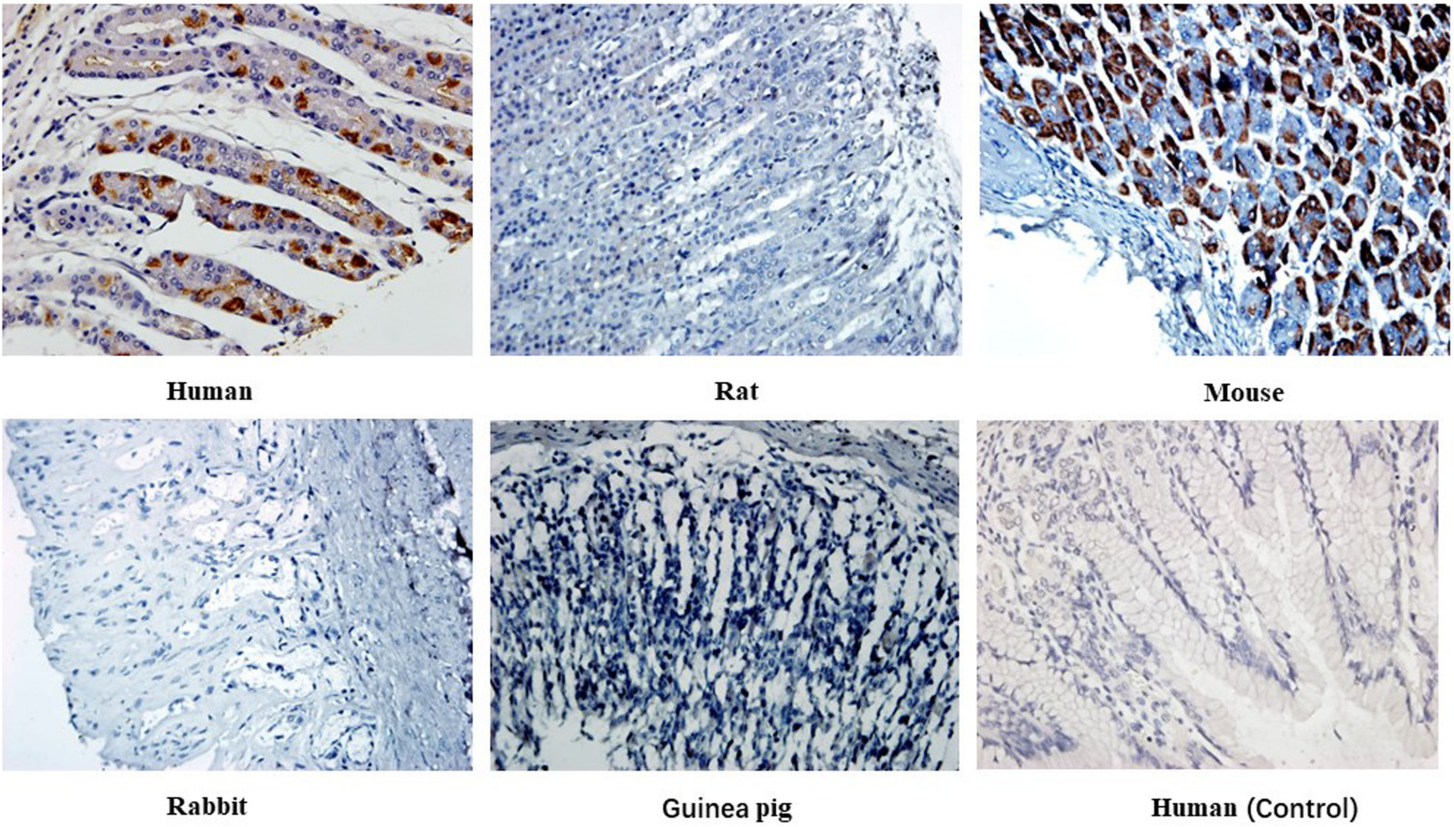
Immunohistochemical detection of the binding characteristics of heterophilic antibodies to stomach tissues from different animals: H7N9-71 antibody was detected by immunohistochemistry with stomach tissues of human, rat, mouse, guinea pig and rabbit respectively. PBS was used as control. Magnification of the image is 400x.

Immunohistochemical staining of 14 heterophilic antibodies that reacted with human stomach, pancreas and kidney tissues with corresponding tissue microarrays from different animal species revealed that these heterophilic antibodies also had different reaction characteristics with different species (**Table 5**). For example, three strains of antibodies, ADV-6, CP-1 and H5N1-32, had different binding characteristics to kidney tissues of different species; ADV-6 reacted only with human kidney tissues, CP-1 reacted with rabbit kidney tissues in addition to human kidney tissues, and H5N1-32 reacted with kidneys of rats, mice, rabbits and human. **Figure 4** showed the results of the reaction of the H7N9-71 antibody with different species of stomach tissues. The above indicated that the distribution of antigens bound by heterophilic monoclonal antibodies is species-specific. Meanwhile, we found that heterophilic antibodies from mice also reacted with the tissues of mice, suggesting that auto-reactive antibodies may also be derived from heterophilic antigens carried by foreign microorganisms.

**Table 5:** Reactivity of the mAbs to different animal species.

mAbs react with	SD rat	Balb/c rat	Guinea pig	Rabbit	Human
Pancreatic					
H1-17	–	–	–	–	+
H1-55	+	+	–	+	+
H7N9-58	+	+	+	+	+
H7N9-59	+	+	+	+	+
H5-67	+	–	–	–	+
Stomach					
H7N9-71	–	+	–	–	+
H7N9-73	–	+	–	–	+
H7N9-77	–	+	–	–	+
H7N9-78	–	+	–	–	+
H7N9-79	–	+	–	–	+
H5-32	–	+	–	–	+
Kidney					
H5-32	+	+	-	+	+
AD-6	-	-	-	-	+
CP-1	-	-	-	+	+

-, Negative; +, Positive.

### 3.6 Serum blocking experiment of patients infected with Epstein-Barr virus

We collected serum from patients with EBV infection to further confirm whether similar heterophile antibodies are produced clinically. Sixty sera from patients with EBV infection were tested using the ELISA method, and five human sera with anti-EBV antibody potencies of 10^-3^ or higher were screened. Immunohistochemical analysis of these 5 sera with normal human tissue microarrays revealed that all 5 patient sera reacted with human testicular tissues, but not with human esophageal and skin tissues, the results were shown in **Table 6**.

**Table 6:** Detection of heterophilic antibodies in sera of patients with EBV infection.

	Testicle	Esophagus	Skin
Serum 8	**+**	**-**	**-**
Serum 30	**+**	**-**	**-**
Serum 31	**+**	**-**	**-**
Serum 37	**+**	**-**	**-**
Serum 45	**+**	**-**	**-**

-, Negative; +, Positive.

**Figure 5:**
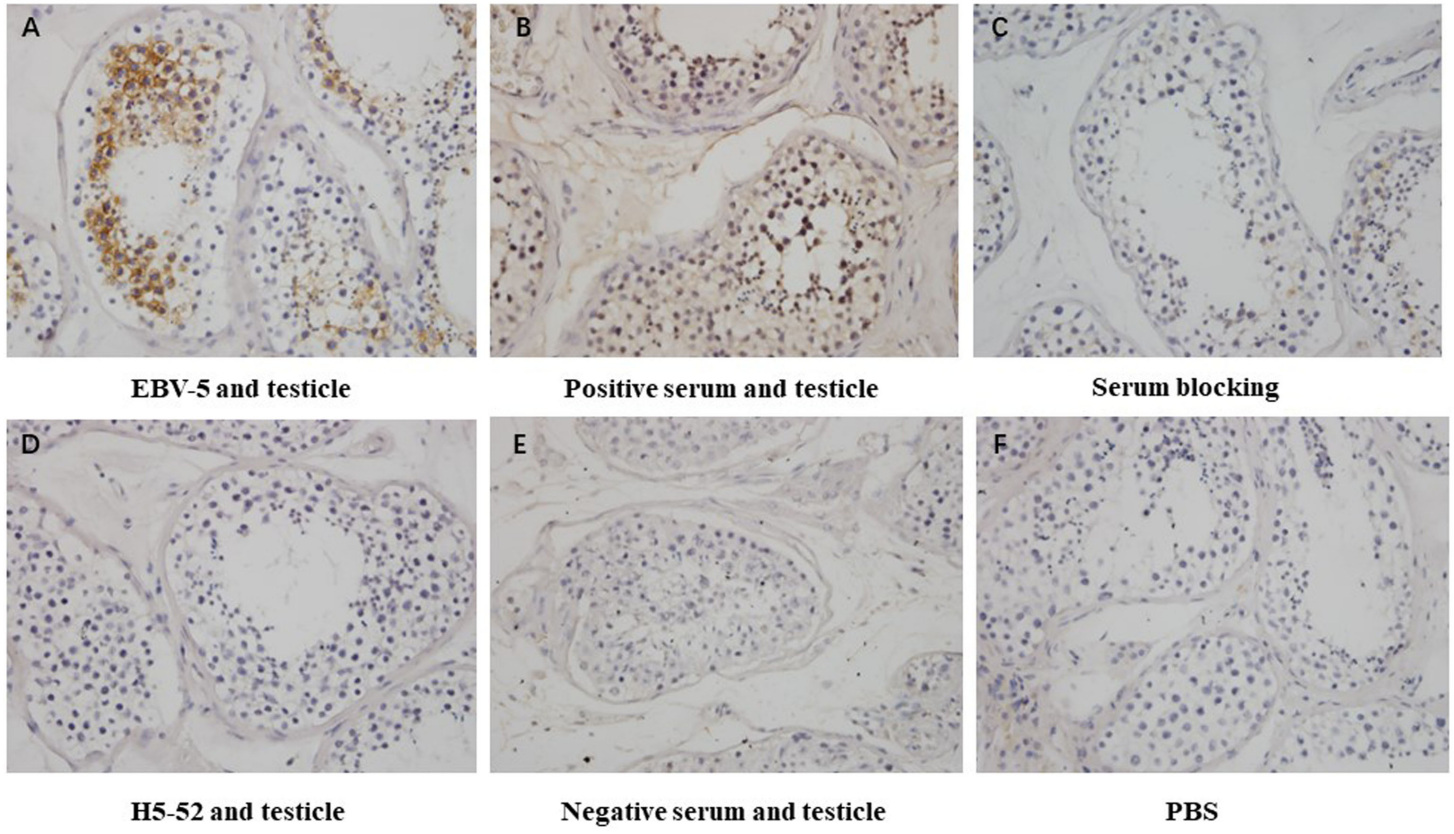
Immunohistochemical detection of the blocking effect of serum on the binding of EBV-5 monoclonal antibody to human testicle: **(A)** shows the reaction of EBV-5 to human testicle, **(B)** shows the reaction of EBV-positive serum to human testicle, **(C)** shows the reaction of EBV-5 to human testicle after blocking with serum, **(D)** shows the reaction of negative antibody to human testicle, **(E)** shows the reaction of negative serum to human testicle and **(F)** is a blank control. Magnification is 400x.

Immunohistochemical methods were used to detect the blocking effect of 5 serum samples of patients infected with Epstein-Barr virus on the binding of 4 strains of heterophilic monoclonal antibodies H1-84, ADV-6, CP-1, EBV-5 to normal testicular tissue. The results showed that the serum of patient 8 blocked the binding of monoclonal antibody EBV-5 to normal testicular tissue, indicating that the EBV-infected patient produced antibodies against the associated heterophilic epitope (**Figure 5**).

## 4 Discussion

Heterophilic antigens are common antigens that exist among humans, animals and microorganisms. A variety of viruses and bacteria have similar antigenic structures with some tissues or extracellular components of normal human body, indicating the existence of heterophilic antigens between microorganisms and human tissues. The immune response to these heterophilic antigens will produce cross-reactive antibodies and cause autoimmune diseases.

Studies have found that the lipopolysaccharide of Campylobacter jejuni is homologous to GM1 of peripheral motor ganglioside and GQ1b of oculomotor nerve. When the body is infected with Campylobacter jejuni, the immune system can produce anti-GM1 and anti-GQ1b cross-reactive antibodies, thus causing Guillain-Barre syndrome (GBS) and Miler-Fisher syndrome (MFS) (9). Antibodies against enterovirus Coxsackie VP1 protein can cross-react with mitochondrial proteins of beta islet cells, which may be associated with infection-induced diabetes (10). Influenza vaccination can significantly increase the incidence of narcolepsy (11, 12). Studies have proved that antibodies against influenza virus nucleoprotein were cross-reacted with human hypoceretin receptor 2, which is present in human brain and is closely associated with brain disorders (13). Recent studies have found that antibodies produced by severe COVID-19 patients will bind to their own proteins, resulting in severe life-threatening symptoms (14). These studies indicated that the existence of heterophilic antigens between microorganisms and human tissues may be one of the mechanisms by which microorganisms induce autoimmune diseases. The above cross-reactions all occurred between microorganisms and specific human tissues, inducing organ-specific autoimmune diseases, but there are also some cross-reactions between microorganisms and multiple tissues. For example, anti-Epstein-Barr virus antibodies can cross-react with nuclear antigens, which are widely found in body tissues, suggesting that the cross-reaction between Epstein-Barr virus and human tissues may be the cause of some systemic autoimmune diseases, such as SLE (15).

Our previous study found that the monoclonal antibody against H1N1 influenza virus HA can cross-react with human islets (16). Furthermore, the microarray containing 33 normal human tissues was used to screen the monoclonal antibody against a variety of pathogenic microbial antigens prepared in our laboratory. The results showed that 21 heterophilic monoclonal antibodies were screened out of 268 strains that could bind to various human tissues. These heterophilic monoclonal antibodies were derived from a variety of pathogenic microorganisms, respectively. This result indicated that heterophilic antigens widely existed between pathogenic microorganisms and human tissues.

Our study also found that the distribution of heterophilic antigens in the body had tissue differences, that is, some heterophilic monoclonal antibody was bound to a single human tissue, while some heterophilic monoclonal antibody was bound to multiple human tissues. These findings suggested that the distribution characteristics of heterophilic antigens may be related to the organ-specific or systemic of autoimmune diseases. Therefore, the study on the tissue distribution of heterophilic antigens will contribute to the understanding of the pathogenesis of autoimmune diseases.

Microbial infections are associated with the development of autoimmune diseases, but not all individuals with microbial infections develop autoimmune diseases. Acute rheumatic fever occurs in 2% to 3% of people with type A streptococcal pharyngitis. During the 2009 global influenza A (H1N1) pandemic, narcolepsy was reported in more than 1300 people who received Pandemrix vaccine (17, 18). Shahed (19) reported that Guillain-Barre syndrome (GBS) was associated with influenza infection, but Kim (20) held the opposite view that the incidence of GBS increased slightly but not significantly during the influenza pandemic in Korea. Chopra (21) highlighted the effect of influenza on the incidence of rheumatoid arthritis, whereas Jain (22) argued that influenza had no effect on the incidence of rheumatoid arthritis. In addition to individual differences due to genetic susceptibility, this controversy may also be caused by individual differences in the distribution of heterophilic antigens. Immunohistochemical staining of pancreatic tissues from 21 different individuals showed that 17 out of 21 individuals (80.9%) had pancreatic tissue bound to H7N9, only two out of 21 individuals (9.5%) had pancreatic tissue bound to H5-67. The same pattern was observed in the immunohistochemistry of stomach and kidney tissues. The differences in the distribution of the above heterophilic antigens may explain why some people develop autoimmune diseases while others do not when infected with the same pathogenic microorganism. These findings will help clinical researchers to assess the risk of autoimmune diseases induced by specific viruses in some individuals, and will help clinicians to better refine clinical treatment plans.

The distribution of heterophilic antigens is not only individually variable, but also species-specific. One study found that infection of turkeys with influenza A virus induced pancreatitis and diabetes in the animals (23). Infection with lymphocytic choroid plexus meningitis virus or polyomavirus stimulates the development of multiple sclerosis disease, with NZB mice being the preferred choice (24). In establishing animal models of SLE, NZB/NZW or NZB/SWR hybrid mice are the best choice (25). In contrast, when establishing animal models of type 1 diabetes, the most commonly used animals are NOD mice (26). The above findings suggest a preference in the selection of animals for animal models of autoimmune diseases, which may be related to the species variability in the distribution of heterophilic antigens. Immunohistochemical staining of tissues from different species with heterophilic antibodies showed that H1-55 reacted not only with human pancreatic tissue but also with pancreatic tissues from SD rats, BalB/c mice and rabbits, but not from guinea pigs. On the other hand, H1-17 reacted only with human pancreatic tissue and not with pancreatic tissue of other species. This result suggested that heterophilic antigens were distributed differently in different species. A good understanding of the species variability in the distribution of heterophilic antigens could help us to better select animals to induce specific animal models of autoimmune diseases.

In this study, the Balb/c murine-derived monoclonal antibodies also exhibited heterophilic reactions with the tissues of Balb/c mice, which was consistent with the results reported by SRINIVASAPPA (27). In addition, the results in **Figure 5** showed that the serum of patients infected with Epstein-Barr virus could block the binding of EBV-5 to human tissues, indicating that patients infected with Epstein-Barr virus produced heterophilic antibodies with the same binding epitope of EBV-5 monoclonal antibody. These results further suggest that autoreactive antibodies may be derived from heterophilic antigens carried by microorganism.

The heterophilic antigens are always present in normal mice, so why does it not elicit an autoimmune response before immunizing with microbial antigens, but stimulation with the microorganism carrying the heterophilic antigens produces heterophilic antibodies? This suggests that there may be a mechanism for the induction of an autoimmune response in the body. By comparing the two states of heterophilic antigens existing on self-protein and microbial protein, it was found that different carriers attached to heterophilic antigens may be the reason for their different effects on the body’s immune system, suggesting that carrier effect may be the main factor affecting the different stimulation effects of heterophilic antigens (28). Therefore, the results of this study suggest that the carrier effect plays an important role in the pathogenesis of infection-induced autoimmune diseases, and the heterophilic antigens carried by microorganism may be the initiator of autoimmune diseases.

In conclusion, the distribution of heterophilic antigens is closely related to autoimmune diseases. The distribution of heterophilic antigens is different in tissue, individual and species. The distribution characteristics of heterophilic antigens can help us to evaluate the susceptibility of autoimmune diseases, predict the development of autoimmune diseases, improve the therapeutic effect of autoimmune diseases and evaluate the prognosis of autoimmune diseases. In addition, further study on the role of carrier effect in the autoimmune response induced by heterophilic antigens has important theoretical and practical value for further understanding of the relationship between microbial infection and autoimmune diseases.

## Data availability statement

The raw data supporting the conclusions of this article will be made available by the authors, without undue reservation.

## Ethics statement

The studies involving human participants were reviewed and approved by the Ethics Committee of Shaanxi Provincial People’s Hospital. The patients/participants provided their written informed consent to participate in this study. The studies involving animals were reviewed and approved by the Biomedical Ethics Committee of Health Science Center of Xi’an Jiaotong University.

## Author contributions

LS: Writing – original draft. NW: Investigation, Writing – review & editing. YF: Methodology, Writing – review & editing. XH: Methodology, Writing – review & editing. QF: Validation, Writing – review & editing. XZ: Validation, Writing – review & editing. YL: Methodology, Writing – review & editing. LY: Funding acquisition, Writing – review & editing. XX: Formal analysis, Writing – review & editing. JH: Writing – review & editing.
