# Supplementary figures and images for: The distribution of heterophilic antigens and their relationship with autoimmune diseases
Source: Front Immunol. 2023 Nov 10;14:1275658. doi: 10.3389/fimmu.2023.1275658 (PMC10667719; doi:10.3389/fimmu.2023.1275658)

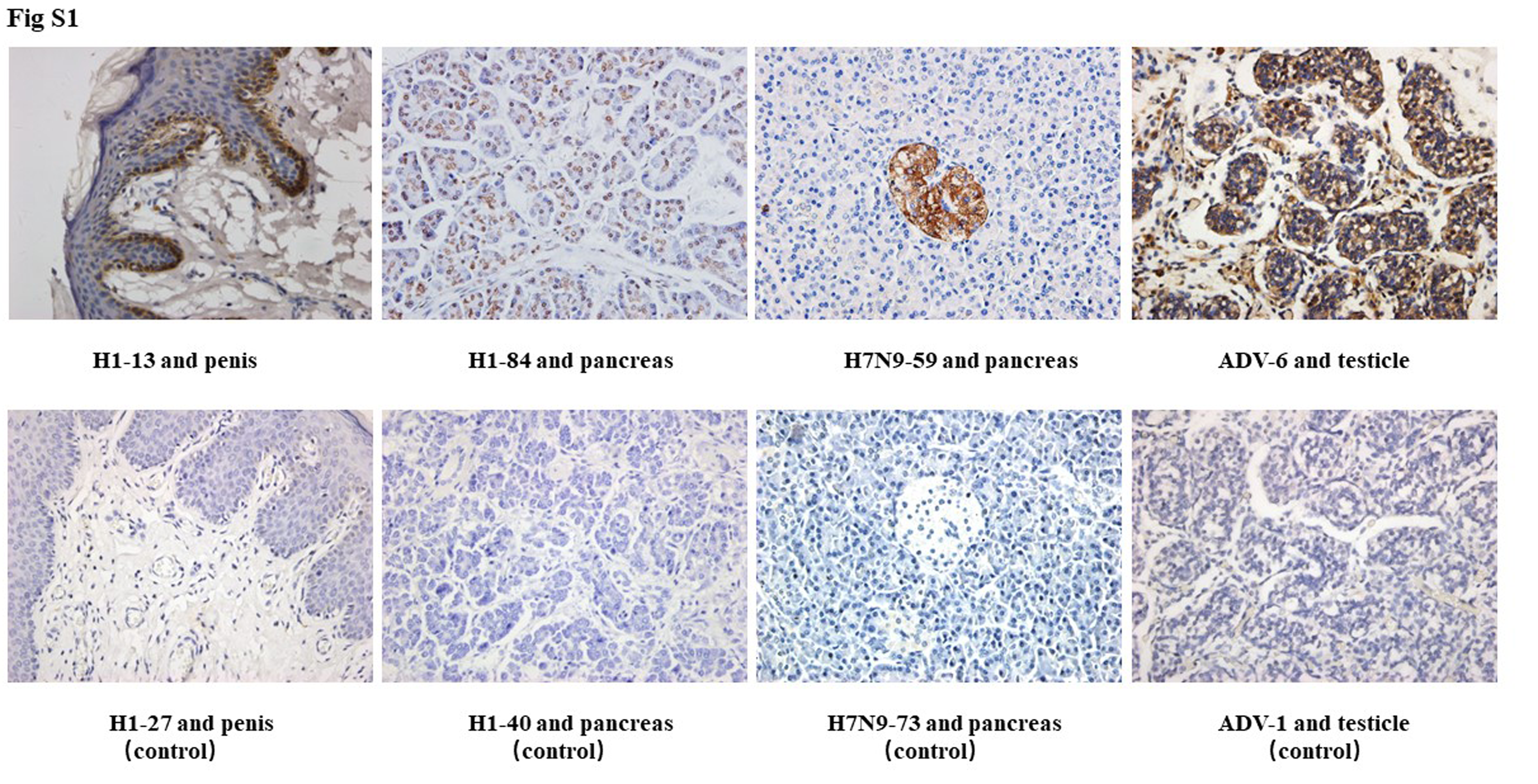

Supplement: Supplementary file 1 [file Image_1.tif]

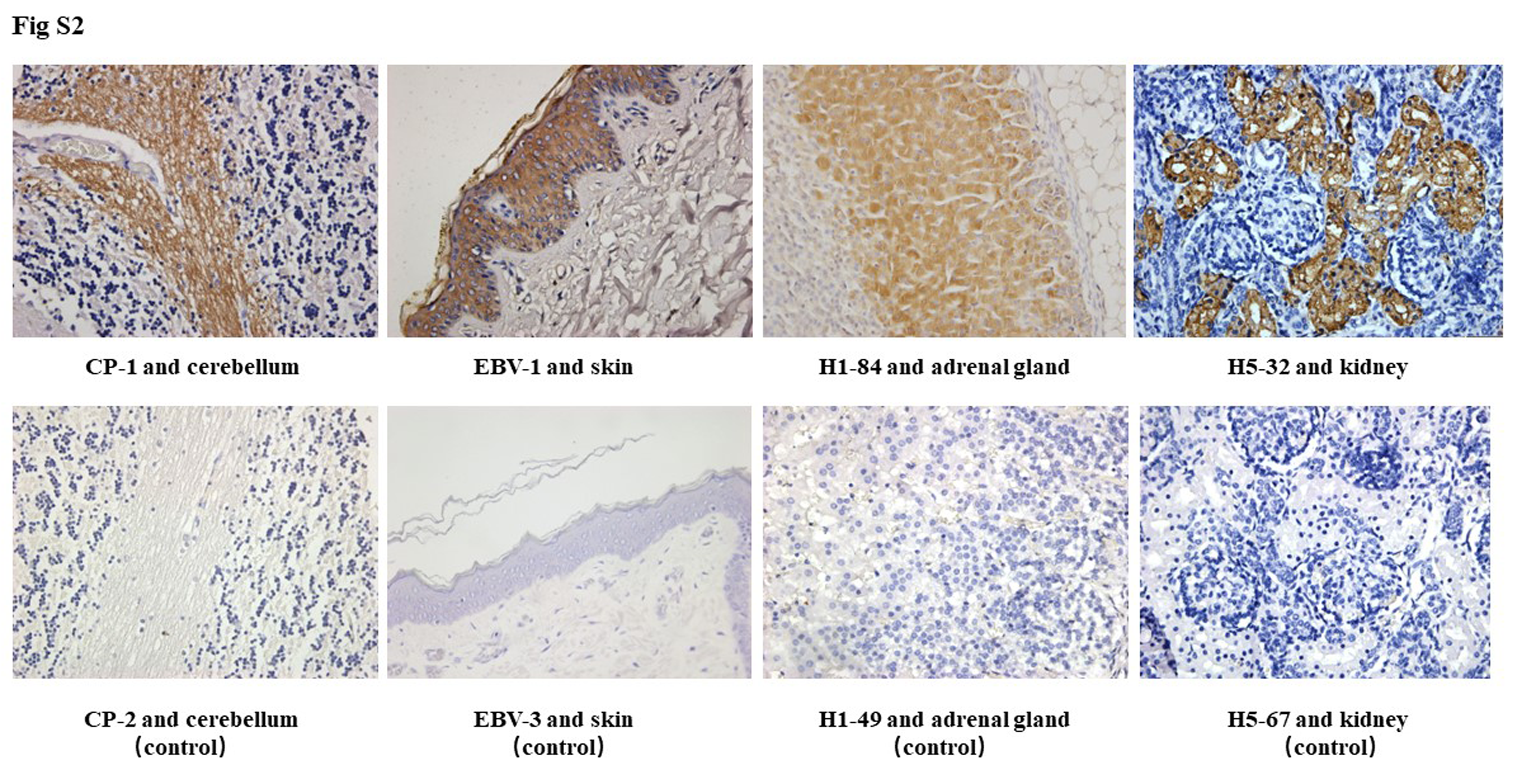

Supplement: Supplementary file 2 [file Image_2.tif]

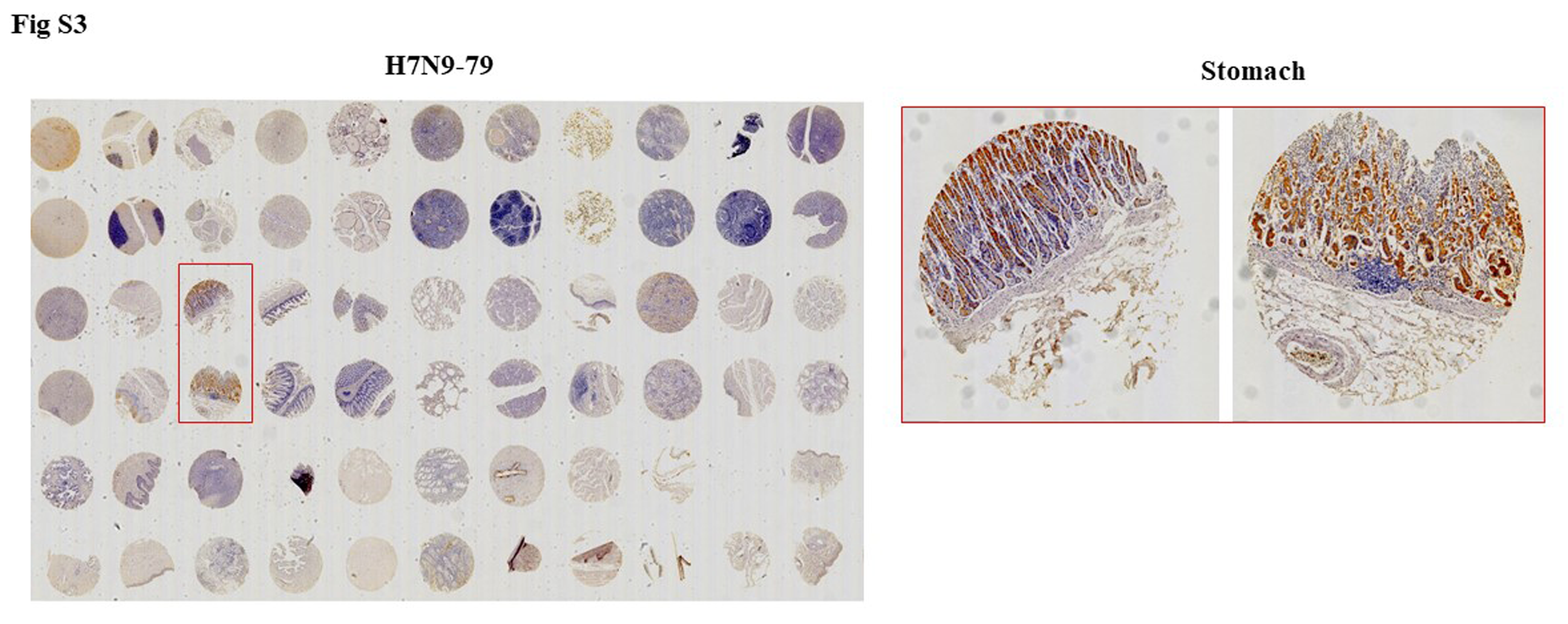

Supplement: Supplementary file 3 [file Image_3.tif]

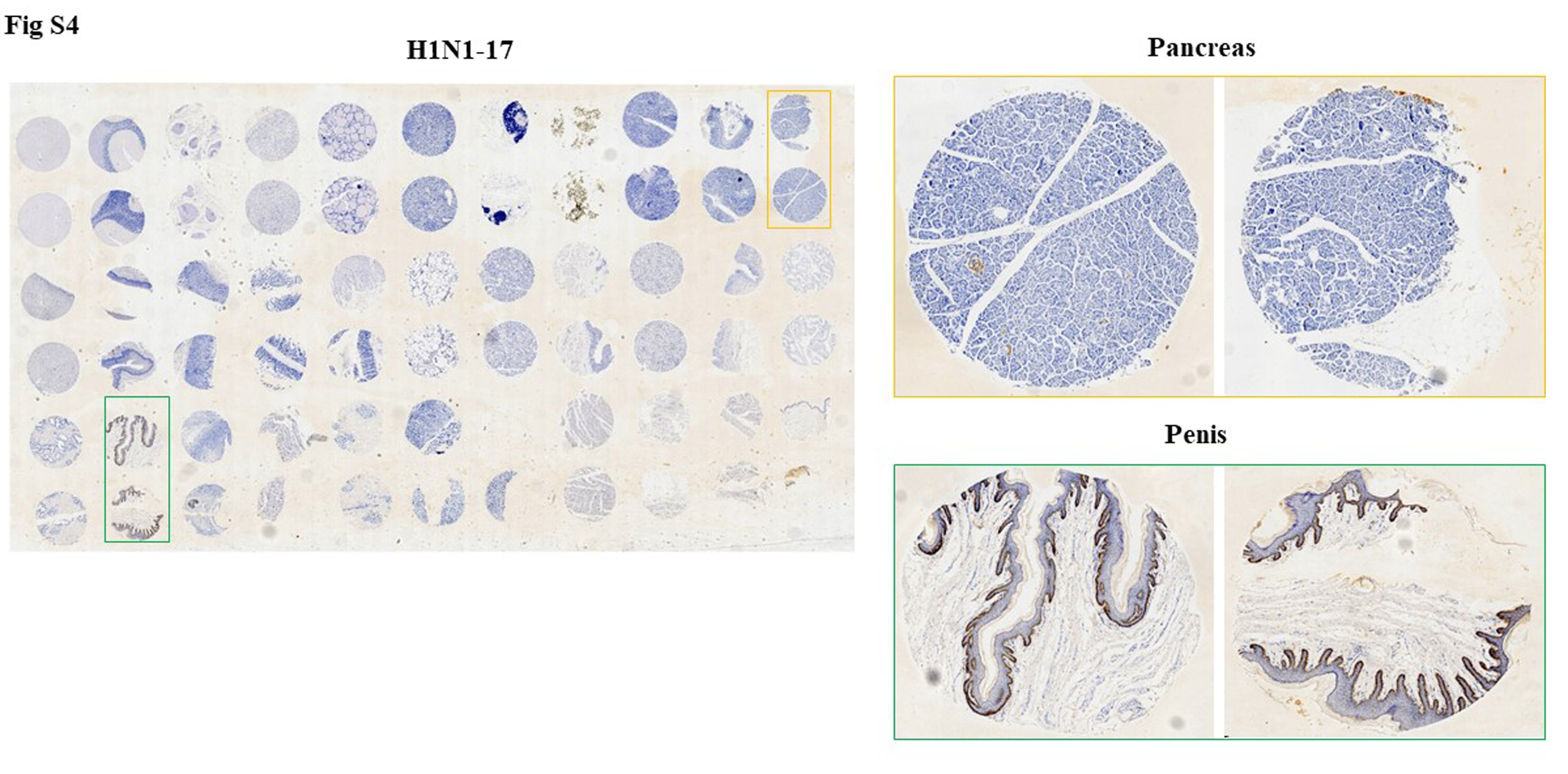

Supplement: Supplementary file 4 [file Image_4.tif]

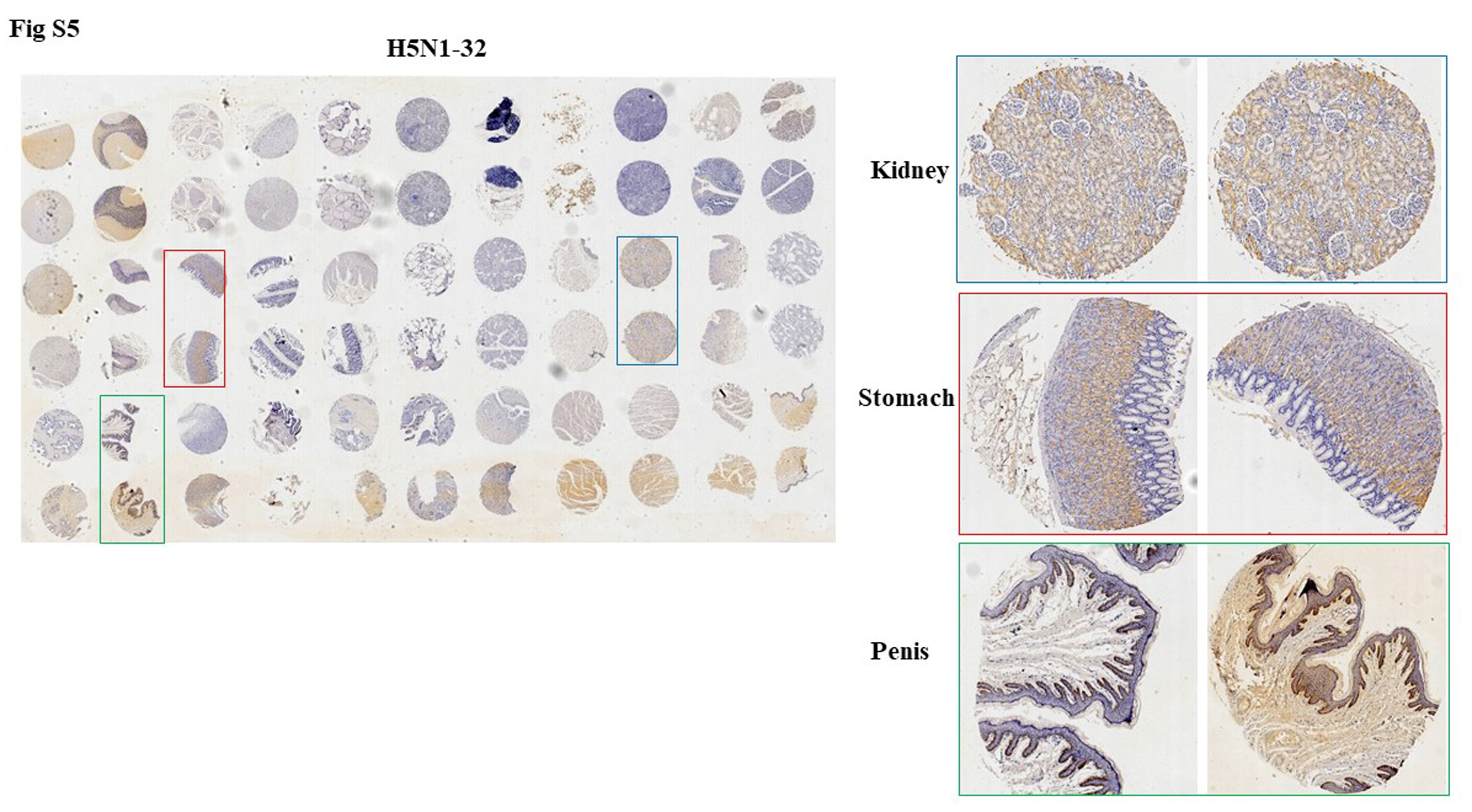

Supplement: Supplementary file 5 [file Image_5.tif]
